# Perspectivism and the Argument from Guidance

**DOI:** 10.1007/s10677-016-9775-9

**Published:** 2016-12-27

**Authors:** Jonathan Way, Daniel Whiting

**Affiliations:** Philosophy, Faculty of Humanities, University of Southampton, Southampton, SO17 1BJ UK

**Keywords:** Normative reasons, Guidance, Objectivism, Perspectivism, Ought, Motivating reasons

## Abstract

Perspectivists hold that what you ought to do is determined by your perspective, that is, your epistemic position. Objectivists hold that what you ought to do is determined by the facts irrespective of your perspective. This paper explores an influential argument for perspectivism which appeals to the thought that the normative is *action guiding*. The crucial premise of the argument is that you ought to φ only if you are able to φ for the reasons which determine that you ought to φ. We show that this premise can be understood in different ways. On one reading, it provides no support for perspectivism. On another reading, the premise lacks support. So, the argument fails. An important upshot of the paper is that the objectivist can embrace the thought about guidance.

## Introduction

*Perspectivists* hold that what you ought to do is determined by your perspective on the facts. *Objectivists* hold that what you ought to do is determined by the facts irrespective of your perspective. What constitutes your perspective? Different answers to this question result in different version of perspectivism. The basic idea, common to them all, is that your perspective is determined by your epistemic position – the evidence you have, or your justified beliefs, or what you know, or what you in a position to know, and so on.

To see the difference, suppose that a drug will cure a doctor’s patient but all her evidence, everything she is in a position to know, etc., suggests that it will kill the patient. According to objectivism, the doctor ought to give the drug. In contrast, according to perspectivism, the doctor ought not to do so. Of course, the case is underdescribed but it is uncontroversial that what you ought to do relative to the facts might diverge from what you ought to do relative to your epistemic position.^1^

Some think that the dispute between objectivists and perspectivists is merely verbal. The facts determine what you ought to do in the ‘objective’ sense, while your epistemic position determines what you ought to do in the ‘perspectival’ sense. But, even if there are different senses of ‘ought’, a dispute remains. In deliberation, we ask ourselves a single question, ‘What ought I to do?’ It is a substantive question whether the ‘ought’ in play here depends on the facts or one’s perspective. This is the question objectivists and perspectivists disagree over (cf. Broome 2013, ch. 2; Kiesewetter 2011; Graham 2010; Lord 2015; Zimmerman 2008: ch. 1).^2^

In this paper, we examine one of the main arguments in support of perspectivism, which we call the *argument from guidance*.^3^ As the name suggests, that argument draws on the compelling, if suggestive, idea that the normative must be able to guide us.

We show that this argument fails. We distinguish two interpretations of the idea that the normative is guiding and argue that, on the interpretation the perspectivist requires, the idea should be rejected, given the principle that *ought implies can*. Not everyone accepts this principle, of course, but it is not open to the proponent of the argument from guidance to reject it, since one of the premises of her argument entails it. The positive lesson that emerges is that, when understood in the right way, the objectivist can embrace the compelling idea that the normative must be able to guide us.

To be clear, we do not here seek to defend objectivism, or to reject perspectivism. Our aim, instead, is to challenge one prominent and influential argument for perspectivism.

## Preliminaries

There are different versions of perspectivism. For simplicity, we focus on a particular kind. Although our central points apply to some other versions of the view, we will not try to show this here.^4^

The version of perspectivism we focus on accepts two familiar ideas. First, what you ought to do is determined by *normative reasons*. Normative reasons are considerations which count in favour of, or against, an action (cf. Scanlon 1998, 17). What you ought to do is determined by how the normative reasons for and against acting weigh up – roughly, you ought to do what the balance of such reasons supports. For instance, if there is a reason for you to take an umbrella, and no stronger reason not to do so, you ought to take an umbrella.

Normative reasons contrast with motivating reasons – the reasons for which you act. In some cases, the reasons for which you act are, or correspond to, reasons for acting. That is to say, in some cases, your motivating reasons are, or correspond to, normative reasons.^5^ For instance, that it is raining might speak in favour of taking an umbrella and be the reason for which you do so. We return below to the connection between normative and motivating reasons.

The second idea that the version of perspective we focus on involves is that normative reasons are *facts*. For example, the fact that it is raining might be a reason to take an umbrella.

Both these ideas are plausible and widely accepted by objectivists and perspectivists alike.^6^ Nonetheless, there is a prima facie tension between them and the perspectivist’s claim that what you ought to do depends on your perspective. Suppose that some fact which does not fall within your perspective counts for some action. For example, it will rain, although you are in no position to know this, you have no evidence that it will rain, and so on. If this reason is weighty enough, it seems that it could make it the case that you ought to take an umbrella. But, in that case, what you ought to do is not a function of your perspective: that it will rain lies outside your perspective.

The perspectivist can avoid this tension in two ways. First, she might say that which facts are reasons for you is itself epistemically constrained. For example, the perspectivist might say that the fact that p is a reason for you only if you know that p, or are in a position to know that p, or have evidence that p, etc. (Dancy 2000, 56–59; Kiesewetter Forthcoming; Markovits 2010, 219; Raz 2011, 111). Second, the perspectivist might say that what you ought to do does not depend on all of the reasons there are but only on the reasons which fall within your perspective. On this view, the fact that p can be a reason for you even if you do not know (etc.) that p. But only facts which you know (etc.) bear on what you ought to do (Lord 2013; Setiya 2014).^7^

The difference between these views turns on whether there are reasons which fall outside your perspective. If we say that a reason which falls within your perspective is a *perspective-relative* reason, the views differ on whether all reasons are perspective-relative. This difference will not be important in what follows. We focus on the thesis which these views share, namely, that what you ought to do is determined by your perspective-relative reasons.

This is an attractive and increasingly popular version of perspectivism, precisely because it respects the plausible ideas that normative reasons are facts and that ‘oughts’ are determined by reasons so understood. Thus although our focus is in this way limited, it is broad enough to be of considerable interest.

For concreteness, we will need to make an assumption about what it takes for a reason to fall within your perspective. Specifically, we will assume that your perspective is constituted by *what you know*:

- P=K That p falls within your perspective if and only if you know that p.

It follows that a reason bears on what you ought to do only if you know the fact which is that reason. We make this assumption solely for presentational purposes. As we will explain, the arguments to follow could proceed given alternative views about what constitutes your perspective.^8^

As mentioned above, we will appeal to the principle that *ought implies can*:

- OIC If you ought to φ, you can φ.

Some objectivists reject OIC, as do some perspectivists. Fortunately, we do not need to defend the principle here. As mentioned above, the proponent of the argument from guidance is committed to it.

In passing, note that whether one should accept OIC turns in part on how ‘can’ is to be understood. We return to this below.

## The Argument from Guidance

Behind the argument from guidance is the intuitive thought that normativity is *action guiding*. In Raz’s words, ‘normative reasons can guide agents’ (2011, 26). As Korsgaard puts it, ‘A practical reason must function […] as a guide’ (2008, 31). In the familiar terminology, normative reasons can be motivating reasons.^9^

You might think that this points toward perspectivism. Reasons tell us what to do, as it were, and they can only do that if we are in a position to listen to and heed their advice. As Gibbons says, since ‘genuinely normative reasons and requirements must be capable of guiding us in some important sense […] these reasons and requirements must be accessible to us in some important sense’ (2013, 132). Reasons must be capable of guiding us in the sense that we are able to act *for* or *on the basis of* those reasons, at least, if they are to determine what we ought to do. And reasons must be accessible in the sense that they fall within our perspective.

These remarks are, of course, suggestive at best. Here is an attempt to make more explicit the line of thought they point to^10^:

1. If you ought to φ, you have the ability to φ for the right reasons, that is, the reasons that make it the case that you ought to φ.
2. If you have the ability to φ for the right reasons, those reasons fall within your perspective.
3. So, if you ought to φ, those reasons fall within your perspective.

If successful, this argument supports perspectivism. Given P=K, its conclusion is that, if you ought to φ, you know the facts which make it the case that you ought to φ.

What is to be said for or against the premises of the argument? That depends on how talk of abilities is to be understood. We will show that the argument from guidance requires a particular interpretation of such talk and that, under that interpretation, premise (1) is unsupported.

## General and Specific Abilities

Suppose that Serena is at the baseline equipped with a ball and racket. In a clear sense, Serena is able to serve – she is in a position to do so. Suppose instead that Serena is on a plane travelling to a match and her equipment is in cargo. Serena is not in a position to serve there and then. Nonetheless, in a clear sense, Serena is still able to serve. Her situation is not like that of a person who has never had the chance to learn tennis or who lacks the strength to raise a racket. For another example, suppose that Gordon knows how to make a cake but lacks the ingredients. In one sense, he is unable to make a cake – he cannot do so here and now. But, in another sense, he is able to make a cake – he has the competence to do so.

Different theorists mark this distinction in different ways.^11^ Following Mele (2002), we will use ‘general ability’ for the sense in which Serena is able to serve even while on the plane, and in which Gordon is able to make a cake even when lacking the ingredients. A general ability to φ is an ability to φ in a wide range of circumstances, if not the present circumstances (here and now). We will use ‘specific ability’ for the sense in which Serena is unable to serve while on the plane, and in which Gordon is unable to make a cake without the ingredients. A specific ability to φ is an ability to φ in the present circumstances (here and now). As the examples illustrate, someone with a general ability to φ might lack the specific opportunity because, for instance, they lack the *opportunity* to φ – as when Gordon lacks the ingredients to make a cake – or because of some kind of *interference* – as when Serena is too tired to lift her racket.

In view of this, (1) is ambiguous between:

- (1g) If you ought to φ, you have the general ability to φ for the right reasons.
- (1s) If you ought to φ, you have the specific ability to φ for the right reasons.

Likewise, (2) is ambiguous between:

- (2g) If you have the general ability to φ for the right reasons, those reasons fall within your perspective
- (2s) If you have the specific ability to φ for the right reasons, those reasons fall within your perspective.

(2g) is clearly false. To be able *in the general sense* to act for a reason of a certain sort, that reason does not need to be within your reach, any more than a tennis racket needs to be within Serena’s reach if she is to be able, in that sense, to serve.^12^

(2s) is plausible and we accept it for present purposes. For example, if the fact that it is raining makes it the case that you ought to take an umbrella, you are not able in the specific sense to take an umbrella when leaving the house on the basis of that fact, that is, for that reason, unless you know that it is raining.

Since (2) must be read as (2s), to avoid equivocation (1) must be read as (1s). Importantly, (1s), unlike (1g), implies OIC. OIC, at least as ordinarily understood, says that ‘ought’ implies specific ability (cf. Vranas 2007).^13^ And, if you have the specific ability to φ for the right reasons, then you have the specific ability to φ.

The claim that reasons must be able to move us is a familiar and plausible one.^14^ It features prominently in debates over the Humean theory of reasons and the possibility of pragmatic reasons for belief. Humeans claim that since reasons must be able to motivate us, and since only considerations appropriately related to our desires can motivate us, only considerations appropriately related to our desires can be reasons (Williams 1981: ch.8). Evidentialists claim that since reasons must be able to motivate us, and since only evidence can motivate belief, only evidence can be a reason to believe (Kelly 2002; Shah 2006).^15^

However, the version of the thought that reasons must be able to move us which features in these debates is weaker than (1s). Humeans claim that even known facts which do not connect to our desires cannot motivate action; evidentialists claim that even known pragmatic considerations cannot motivate belief. In both cases, the claim is that we lack the *general* ability to be moved by a certain sort of consideration. This is a claim objectivists can accept.

So far then, it seems open to objectivists to say that (1g) is all that we need to capture what is plausible about (1). And since (2g) is false, the argument from guidance fails under this interpretation.

## The Argument from Creditworthiness

Lord (2015) argues in support of premise (1). Although he does not note the distinction above, we will consider whether the arguments he provides support (1s).^16^

Lord appeals to a notion of *creditworthiness*. You are creditworthy or deserve credit for something when it reflects well on you and insofar as you are responsible for it. In this sense, you can be creditworthy for your achievements, ideas, decisions, traits, and so on. Among the things you can be creditworthy for, and the thing Lord focuses on, is doing what you ought to do. Famously, you can do what you ought to do without being creditworthy for doing so. When Kant’s shopkeeper refrains from overcharging an inexperienced customer, he does what he ought to do but, according to Kant, he is not creditworthy for doing the right thing if he does it for reasons of profit rather than fairness (1998, 4:397). Or to adapt our earlier example, if a doctor ought to give her patient a drug because it will save the patient’s life, but does so on the grounds that it will free up some space in her pockets, she does what she ought to do but is not creditworthy for having done so.

What is required to be creditworthy for doing what you ought to do? According to a popular and plausible view, you deserve credit for doing what you ought to do just in case you do it for the right reasons, that is, for the reasons which make it the case that you ought to φ (see Arpaly 2002; Markovits 2010). For instance, if the doctor gave the patient the drug for the reason that it will save the patient’s life, the doctor would be creditworthy for doing what she ought to do – giving the patient the drug.

With this background, we can state Lord’s argument for (1s). If (1s) is false, there will be cases in which a subject ought to φ even though she is not able (in the specific sense) to get credit for φing. Suppose that the doctor ought to give her patient a drug but does not know the facts which make it the case that she ought to do this. In that case, she is not able to give the drug for the right reasons. So, she is not able to get credit for giving the drug.

Lord takes this to be implausible. It cannot be the case that you ought to perform some act if you are not able to do so in a way that is creditworthy. So, (1s) must be true.

## Ought Implies Can

The central assumption in this argument is:

- Credit You ought to φ only if you have the specific ability to φ in a way that is creditworthy.

One might doubt this assumption. That we cannot always get credit for doing the right thing is, one might think, an unfortunate fact of life, not a strike against objectivism. Rather than pursue this directly, we will argue that, given OIC, there will be cases in which a subject ought to perform some act but can only do so in a way which is not creditworthy. The perspectivist who accepts OIC should thus reject Credit. In that case, the perspectivist is left without this argument for (1s), hence, for (3).

Consider:

DOCTOR A doctor is deciding whether to give drug A or drug B to a patient who has a painful and fatal disease. She knows that A will completely cure the patient, relieving all the patient’s suffering and saving her life, and that B will not save the life of her patient but will relieve the patient’s suffering. The doctor also knows that, if she tries to give one drug, she will be unable to give the other. However, though she is no position to know this, and despite evidence to the contrary, the doctor is unable (in the specific sense) to give A. As it happens, the doctor gives B to the patient for the reason that it will relieve her suffering.^17^

Given OIC, both objectivists and perspectivists should agree that it is not the case that the doctor ought to give A, since she is unable to give A. What then ought she to do? If we also assume that there are no facts which count against giving B, it is plausible that the fact that giving B will relieve the patient’s suffering is a decisive reason to give B, and thus that she ought to give B. But the doctor is not creditworthy for giving B. So, given OIC, DOCTOR is a counterexample to Credit: the doctor ought to give drug B but lacks the specific ability to do so in a creditworthy fashion.^18^

We take it to be intuitive that the doctor is not creditworthy for giving B, indeed, that she is not able (in the specific sense) to give B in a creditworthy fashion. To bolster this point, note that, in giving B when it seems from her perspective that she can give A, the doctor seems to be manifesting callous indifference to the life of her patient. To be creditworthy in giving B the doctor would have to have some reason to think that she cannot give A (or lack reason to think that she can give A). But she does not.

Here is another way of arguing against Credit by appeal to DOCTOR. Given OIC, it is not the case that the doctor ought to give A. Given Credit, it is also not the case that she ought to give B. So, OIC and Credit together imply that there is nothing the doctor ought to do in this case. But this claim is extremely counterintuitive. After all, the doctor has a third option: she can do nothing. If it is not the case that the doctor ought to give B, she must be permitted to give B and also permitted to do nothing. But it is counterintuitive that the doctor is permitted to do nothing, when she knows that she could instead relieve the patient’s suffering. And since giving B is the only permitted option, given OIC, then she ought to give B, contra Credit.

We conclude that, given OIC, we should reject Credit. In that case, Lord’s argument from creditworthiness for (1s) fails, and so the argument from guidance in support of perspectivism fails. Perhaps there are other considerations which favour (1s) than those relating to creditworthiness. But the onus is on the perspectivist to provide them.^19^

As mentioned above, rejecting OIC instead of Credit is not an option available to the proponent of the argument from guidance, since (1s) entails OIC.

Also, as noted above, the objection does not turn on P=K. Since the doctor knows the facts which constitute the reasons in this case, she will meet any weaker constraint on what it takes for a fact – hence, a reason – to fall within a person’s perspective (for example, rational true belief). If someone proposes a stronger constraint on what it takes for a fact – hence, a reason – to fall within a person’s perspective, we can revise the case so that the doctor meets it. For example, if someone proposed that a fact falls within a person’s perspective only if that person knows that she knows that fact, we can revise the case so that the doctor knows that she knows that drug A will completely cure the patient, and so on.

## Replies

There are two ways for a proponent of the argument from guidance to respond to the above case – by insisting that the doctor is creditworthy or by denying that she ought to give B. We consider these in turn.

### Partial Credit

One might concede that the doctor is not wholly creditworthy – deserving of *full* credit – but insist that she is nonetheless creditworthy to some degree – deserving of *partial* credit. After all, the doctor is manifesting some concern for her patient’s suffering. So, the above case does not undermine the following:

- Credit* You ought to φ only if you have the specific ability to φ in a way that is partially creditworthy.

However, Credit* does not support (1s). To see this, suppose that a person ought to φ due to a host of reasons, most of which lie outside of her perspective, one of which falls within it. She φs for the reason of which she is aware. One might think that the person deserves some credit for φing – since she φs for one of the reasons which make it the case that she ought to φ – but not full credit – since she φs in ignorance of the bulk of the considerations which determine that she ought to φ. This case satisfies Credit*. So, Credit* only supports:

- (1s*) If you ought to φ, you have the specific ability to φ for some of the right reasons.

(1s*), in turn, only supports a weak version of perspectivism, according to which some but not all of the relevant reasons must fall within your perspective.

This is not an attractive version of perspectivism and we doubt that it will appeal to perspectivists, since, as just noted, it allows considerations outside of a person’s perspective to play, not just a role, but a dominant role in determining what she ought to do. As a result, it will not deliver perspectivist-friendly verdicts in many cases.^20^

So, the appeal to the idea of partial credit does not help the proponent of the argument from guidance to reach her desired conclusion, namely, (3).

### Trying

An alternative response to DOCTOR, and in support of Credit, is to deny that the doctor ought to give B. Proponents of this response must say what it is that the doctor ought to do instead of giving B. This alternative must be something she is able (in the specific sense) to do, and in a creditworthy fashion. We take it that the most plausible version of this response says that the doctor ought to *try* to give A.^21^

One problem for this response is that we can revise DOCTOR so that the doctor is not even able (in the specific sense) to try to give A. In this version of the case, there are no acts, physical or mental, that she can perform that would constitute trying to give A.^22^ Moreover, we can add that, by the time the doctor realises that she cannot try to give A, the opportunity to give B has passed. Given OIC, if the doctor cannot try to give A, it is not the case that she ought to do so. Since doing so will help the patient, she ought instead to give B. But, if the doctor gives B, she is not creditworthy.

One might object that, for any act, a person can (in the specific sense) try to perform it. But that is simply false. To try to perform an act is itself to perform some (possibly mental) act and, like any other act, a person might lack the specific ability to perform it. Compare: a loving parent cannot try to kill her children. Even if she has the general ability to perform acts of that type, the parent’s love prevents her from exercising it in her circumstances; that is, her love interferes with her general ability to make attempts on her children’s lives. Compare also: a mathematician has the general ability to solve a difficult math problem but cannot in her present circumstances so much as try to do so, since she is deeply depressed, say, or preoccupied with lecturing. In a similar fashion, even if the doctor is in general able to try to give A, something might interfere with that ability in the case at hand.

It might be replied that, even if the doctor cannot try to give A, there will still be other options available to her. For instance, perhaps she can try to try to give A, or perhaps she can try to find out whether she can give A. It might then be suggested that the doctor ought to take one of these options, and that she has the specific ability to do so in a creditworthy fashion.

However, it is not clear why there must always be some such option available. Just as it can be impossible for a person to try to do something, it can be impossible for that person to try to try to do it, to try to find out whether she should do it, or to do anything else which appears to be a means to doing it. To continue with one of the examples above, a parent’s love might make it impossible for her, not just to try to kill her children, but to try to try to do so, or to try to find out how to do so, and so on. We can stipulate that the doctor lacks any such options with respect to giving A.

It is also worth noting that both of these suggestions face independent problems. A problem with the first is that it is not clear that it is even possible to try to try to do something (cf. Clarke 2009, 347).^23^ To the extent that we can make sense of it, trying to try to do something seems to be a way of trying to do that thing. A problem with the second is that it is not clear which facts in the doctor’s perspective decisively support trying to find out whether she can give A. The most plausible candidate for a fact which favours trying to find out whether she can give A is the fact that she does not know whether she can give A. But this is not a fact that falls within the doctor’s perspective.

### Still Trying

Although we think that the revised case succeeds in undermining Credit, we need not rely on it. There is an additional problem facing the perspectivist who claims that, in the original case, the doctor ought to try to give A.

If the doctor ought to try to give A, then the balance of reasons supports doing so. The perspectivist taking this line must thus identify the reasons that support trying to give A. This is not straightforward. In many cases, reasons to try to act derive from reasons to act. For instance, a teacher’s reasons to try to finish marking tonight derive from the reasons to finish marking tonight. Since there are no reasons to give A, there are no reasons of this sort for the doctor to try to give A.^24^

The most promising suggestion here is that the reason to try to give A is provided by the *expected value* of doing so.^25^ This idea is naturally combined with a general picture on which the considerations within a person’s perspective – that is, what she knows – provide reasons for attempts by providing evidence that those attempts will realise or promote something of value. The expected value of an attempt is a function of the relevant value and the likelihood that the attempt will succeed in promoting or realising it.^26^

We do not object to this picture. But it does not help the perspectivist’s defence of Credit. To see this, consider the following version of the case.^27^ The value of giving A, that is, of saving the patient, is 1, while the value of giving B, that is, of relieving her suffering, is 0.8. Relative to what the doctor knows about her situation, the probability that she can give B is 1. So, the expected value of trying to give B is 0.8. Relative to what the doctor knows, the probability that she can give A is 0.7. So, the expected value of trying to give A is 0.7. Given the above picture, the doctor ought to try to give B. However, the doctor (falsely but reasonably) believes that she can give A. If she tries to give B, she is not creditworthy for doing so. On the contrary, it seems callous not to attempt to give A. So, even if we grant that the expected value of trying provides reasons for doing so, there remain counterexamples to Credit.

One might object that it is not callous for the doctor to try to give B in this version of the case. After all, trying to give B has a higher expected value than any of her alternatives. How could it be callous to be guided by expected value?

The crucial point is this. The expected value of giving B, on the above conception of expected value, is relative *only to the known facts*. However, when thinking about whether or not a course of action is callous, we do not only take into account the known facts, but also what the agent reasonably believes, truly or falsely. Once we take this into account, it is clear that the doctor would be callous to try to give B in this revised case. Since the doctor reasonably believes that she can give A, and knows that A will save the patient’s life, she shows indifference to the patient’s life unless she tries to give A.

It might now be replied that it cannot be reasonable for the doctor to believe that she can give A when this is only 0.7 probable on her knowledge. But similar considerations apply here too. What is reasonable for a person to believe does not depend only on what she knows but also on her other reasonable beliefs, true or false, perceptual experiences, veridical or non-veridical, and so on. So, what makes it reasonable for the doctor to believe that she can give A need not be something she knows.^28^^,^ 29

One might now wonder whether the perspectivist’s problems stem from the assumption that one’s perspective is constituted by what one knows, that is, by P=K. It might seem that the perspectivist can avoid these problems if she takes the more liberal view that a person’s perspective is constituted by her reasonable beliefs.

However, since we can have reasonable false beliefs, this more liberal view is incompatible with the popular and plausible assumption that reasons are facts. It thus lacks one of the central attractions of the version of perspectivism we have been focusing on. Furthermore, it is not clear that even this move will help the proponent of the argument from guidance. For it is arguably callous for the doctor to try to give B if she so much as believes that she can give A – reasonably or not. We can thus set up a version of the case in which the expected value of trying to give B relative to the doctor’s reasonable beliefs is greater than the expected value of trying to give A, and yet in which the doctor lacks the specific ability to try to give B in a creditworthy fashion because she unreasonably believes that she can give A.

We conclude that the proponent of the argument from guidance cannot plausibly maintain that the doctor ought to try to give A, at least in the revised version of the case discussed in this section. This attempt to defend Credit from our counter-example thus fails.

## Conclusion

The crucial premise in the argument from guidance is:

1. If you ought to φ, you have the ability to φ for the right reasons, that is, the reasons that make it the case that you ought to φ.

We have argued that (1) can be understood in different ways. On one interpretation, the one the perspectivist needs, the premise lacks support. We argued against the case for it by appeal to the principle that *ought implies can* (§§6–7). On reflection, it may be unsurprising that a major line of thought leading to perspectivism is in tension with that principle. After all, perspectivism says that what you ought to do is determined by your perspective. Whether or not you can do something need not fall within your perspective.^30^ Unfortunately for the proponent of the argument from guidance, rejecting the principle is not an option.

On the other interpretation of the relevant premise, as discussed above, the objectivist can accept it (§4). A person ought to perform some act only if she is able in the general sense to do so for reasons of the appropriate sort. In this sense, the objectivist can agree that the normative is action guiding.

## Footnotes

1 In this paper, we focus on the dispute between perspectivists and objectivists concerning how you ought to *act*. There is a parallel dispute concerning what you ought to believe. (For discussion, see Feldman 1988; Gibbons 2013; Littlejohn 2012; Way and Whiting 2016a)

2 Not everyone accepts this line of thought (cf. Sepielli Forthcoming) but it is not our aim here to defend it. Our concern is with a dispute among those who do accept it.

3 Another influential argument for perspectivism appeals to situations of structural uncertainty (see Dancy 2000, 57–58; Jackson 1991; Kiesewetter 2011; Lord 2015; Regan 1980; Ross 2012; Setiya 2014; Zimmerman 2008, ch. 1). Since this argument has already had much attention (see, for example, Graham 2010; Kiesewetter Forthcoming; Sepielli Forthcoming), we will not discuss it here.

4 We will not, for example, discuss Zimmerman’s (2008) perspectivism.

5 For discussion, see Alvarez 2010; Dancy 2000.

6 Objectivists who accept them include Parfit (2011) and Schroeder (2007). Perspectivists who accept them include Broome (2013), Dancy (2000, 56–59), Kiesewetter (forthcoming), Kolodny (2007), Lord (2013), Markovits (2010), Raz (2011, 211), Scanlon 1998 (see 2008, ch.2 for his perspectivism), and Setiya (2014).

7 Several philosophers seem to accept both perspectivism and the familiar ideas but do not explain how these are to be reconciled (see, for example, Broome 2013; Kolodny 2007; Scanlon 2008, ch. 2). Although he does not accept that reasons are facts, Gibbons (2013) defends a view which is otherwise similar to the first approach.

8 As noted, perspectivists differ on what constitutes one’s perspective. Kiesewetter (forthcoming) endorses the left-right direction of P=K but leaves open whether the right-left direction holds. Lord (2013, ch. 3) and Dancy (2000, 56–59) defend versions of the view that your perspective is what you are in a position to know.

9 This thesis is often called ‘internalism’. So understood, internalism should be distinguished from the view – also sometimes called ‘internalism’, and which below we call the ‘Humean theory of reasons’ – that all reasons depend on our desires. Given this diversity of usage, we prefer to avoid the term ‘internalism’. We discuss the connection between the argument from guidance and an argument for the Humean theory below.

10 Such lines of thought date back at least to Prichard. According to Prichard, if duty ‘depends on certain facts of the situation’, there will be cases in which, ‘though we may have duties, we can never, strictly-speaking, do a duty […] *because* it is a duty’. This shows, he continues, that one’s duty depends ‘on our being in a certain attitude of mind towards the situation in respect of knowledge, thought, or opinion’ (2002, 89). For recent versions of the argument, see Dancy 2000, 59; Gibbons 2013, ch. 6; Lord 2015; Raz 2011, 109–111. The version we discuss here is closest to Lord’s.

11 See, for example, Clarke 2009; Honoré 1964; Kenny 1975; Maier 2015; Mele 2002; Whittle 2010.

12 This point does not beg the question against the perspectivist who holds that all reasons are perspective-relative (in the sense explained above). Suppose that it is raining but you do not know this. If all reasons are perspective-relative, the fact that it is raining is not a reason for you to take an umbrella. Nonetheless, you still have the general ability to do things such as taking an umbrella on the basis of that kind of consideration. Were you to learn that it is raining, hence, were there to be a reason for you to take an umbrella, you could do so for that reason.

13 If OIC only concerned general ability, then it could not explain why, for instance, it is not the case that Serena ought to serve when she is on the plane and her racket is in cargo.

14 For relevant discussion, see Way and Whiting 2016b.

15 To say that a belief is motivated by a reason is here to say that it is held for that reason.

16 Lord offers two arguments in support of (1). Since the arguments are closely related, and the points we make apply to both, we focus our attention on the argument from creditworthiness, confining comments on the further argument to n18.

17 It is no part of the case as described that the doctor believes that she ought to give A. Perhaps she has no belief about what she ought to do. Perhaps she believes that she ought to give B. Such details are irrelevant to the points that follow.

18 It is not intended as a counterexample to the claim that you deserve credit for doing what you ought to do just in case you do it for the right reasons. A proponent of that view might deny that the doctor is acting for the right reason *as such*, since she is insensitive to its being the right reason. Alternatively, she might claim that the doctor is *partially* creditworthy. We discuss partial credit below.

19 Lord’s other argument for (1), understood here as (1s), rests on: No Accident You ought to φ only if you have the specific ability to φ and thereby non-accidentally do what you ought to do. Lord argues that non-accidentally doing what you ought to do requires that the reasons which determine what you ought to do fall within your perspective. If they do not, you can only do what you ought to do for the wrong reasons or for no reasons at all. In that sense, it would be a happy accident if you did the right thing. So, if (1s) is false, there will be cases in which you are not able to non-accidentally do what you ought to do. However, it should be clear that in this sense the doctor in DOCTOR is only able to do the right thing accidentally. So, given OIC, this case shows that No Accident is false too. There are, of course, replies to these claims. Those replies will mirror those we consider below in relation to the argument from creditworthiness. So, for the sake of brevity, we will not rehearse them here.

20 For example, cases of structural uncertainty (cf. fn. 3).

21 The proponent of the argument from guidance might attempt to bolster their denial that the doctor ought to give B by appealing to a principle of *luminosity*: that you ought to φ only if you have the specific ability to know that you ought to φ. Since it seems clear that the doctor does not have the specific ability to know that she ought to give B – after all, she reasonably thinks she can give A – luminosity implies that it is not the case that she ought to give B. However, luminosity is highly questionable. Following Williamson (2000), many deny that any non-trivial condition is luminous. Quite independently of that, not being able to tell what you ought to do is a familiar, if frustrating, experience. In any case, proponents of luminosity will still need to say what it is that the doctor ought to do in the above case. It is this issue we focus on here.

22 Or, for that matter, trying to try to give A, or trying to try to try to give A, and so on.

23 As Clarke observes, it is possible to try to bring it about that you try to do something – for example, by providing yourself with an incentive for trying to do it. But trying to bring it about that you try should be distinguished from trying to try.

24 This assumes the principle *reason implies can*: RIC If you have a reason to φ, you can φ. Here ‘can’ is understood to ascribe specific ability. We take RIC to be independently plausible. It is also very natural for proponents of OIC, which includes proponents of the argument from guidance, to accept the principle. For one thing, it offers a simple explanation for why OIC holds. For another, denying RIC has awkward consequences – for instance, that you can have most reason to do something that it is not the case that you ought to do. For defence of RIC, see Streumer 2007; Vranas 2007

25 There can also be other kinds of reasons to try to do things you cannot do. For instance, reasons to try can be provided by the benefits of trying, or by its expressive or symbolic value. However, we can stipulate that there are no benefits in DOCTOR. There may be some expressive value – in trying to give A, the doctor shows care for the patient. But it seems doubtful that this provides strong enough reason to outweigh the reasons to give B, thereby saving the patient’s life.

26 For a view of this sort, see Broome 2013, ch. 3. For a related view, see Zimmerman 2008, ch. 1.

27 We make some simplifying assumptions about the units and functions involved but for illustrative purposes only.

28 Note that we could also adjust the numbers to avoid this concern.

29 This implies that what it is reasonable to believe is not determined solely by one’s reasons. But given the assumption that one’s reasons are what one knows, and the very plausible claim that one’s reasonable but false beliefs bear on what it is reasonable to believe, it is clear that perspectivists of the sort we are here concerned with must reject this assumption.

30 This point is noted (in different contexts) by Graham (2010) and Kiesewetter (2011).
